# Prediction of bone metastasis risk of early breast cancer based on nomogram of clinicopathological characteristics and hematological parameters

**DOI:** 10.3389/fonc.2023.1136198

**Published:** 2023-07-14

**Authors:** Zhaokun Tian, Chao Li, Xinzhao Wang, Haiyin Sun, Pengyu Zhang, Zhiyong Yu

**Affiliations:** Breast Cancer Center, Shandong Cancer Hospital and Institute, Shandong First Medical University and Shandong Academy of Medical Sciences, Jinan, China

**Keywords:** bone metastasis, nomogram, predictive model, breast cancer, clinicopathological characteristics, hematological parameters

## Abstract

**Objectives:**

The purpose of this study was to determine the independent risk factors for bone metastasis in breast cancer and to establish a nomogram to predict the risk of bone metastasis in early stages through clinicopathological characteristics and hematological parameters.

**Methods:**

We selected 1042 patients with breast cancer from the database of Shandong Cancer Hospital for retrospective analysis, and determined independent risk factors for bone metastatic breast cancer (BMBC). A BMBC nomogram based on clinicopathological characteristics and hematological parameters was constructed using logistic regression analysis. The performance of the nomograph was evaluated using the receiver operating characteristic (ROC) and calibration curves. The clinical effect of risk stratification was tested using Kaplan-Meier analysis.

**Results:**

BMBC patients were found to be at risk for eight independent risk factors based on multivariate analysis: age at diagnosis, lymphovascular invasion, pathological stage, pathological node stage, molecular subtype, platelet count/lymphocyte count, platelet count * neutrophil count/lymphocyte count ratio, Systemic Immunological Inflammation Index, and radiotherapy. The prediction accuracy of the BMBC nomogram was good. In the training set, the area under the ROC curve (AUC) was 0.909, and in the validation set, it was 0.926, which proved that our model had good calibration. The risk stratification system can analyze the risk of relapse in individuals into high- and low-risk groups.

**Conclusion:**

The proposed nomogram may predict the possibility of breast cancer bone metastasis, which will help clinicians optimize metastatic breast cancer treatment strategies and monitoring plans to provide patients with better treatment.

## 1 Introduction

Breast cancer (BC) is the most common malignancy and the leading cause of cancer-related deaths worldwide in women (1). Despite remarkable developments in the systemic treatment of BC, it remains an incurable disease at advanced stages, and the clinical symptoms of metastatic breast cancer (MBC), which exhibit a high degree of heterogeneity, are extremely diverse. The most frequent metastatic location of BC is the bone. Seventy percent of patients with stage IV BC have bone metastases (2), and 17–37% of patients have only bone metastasis (3–5). Patients with bone metastatic breast cancer (BMBC) have an average overall survival of 40 months (6), a 3-year survival rate of 25%, and a 5-year survival rate of 13% (7). Furthermore, adverse skeletal-related events such as hypercalcemia, nerve root or spinal cord compression, fractures, and pain, are frequently caused by bone metastases, and they have a significant negative impact on BC (6).

Distant metastasis-free survival (DMFS) may be affected by clinicopathological factors, including lymphovascular invasion (LVI), Ki-67 expression, human epidermal growth factor receptor 2 (HER-2) status, estrogen (ER) or progesterone (PR) status, lymph node stage, and tumor size. Moreover, hematological indicators, such as platelet count/lymphocyte count ratio (PLR), neutrophil count/lymphocyte count ratio (NLR), and monocyte count/lymphocyte count ratio (MLR), have sufficient prognostic value for the recurrence of several cancers, including gastric cancer (8–10). The efficacy of treatment, and treatment biomarkers (circulating immune cells) have been discussed in previous research. Moreover, prognosis, and survival from cancer have been studied (11–13). Furthermore, the Glasgow Prognostic Score and Systemic Immunological Inflammation Index (SII), which represent the patient’s immune status and degree of inflammation, have been proposed as predictive tools in patients with cancer (14, 15). In clinical practice, it is generally believed that an increase in inflammatory markers in the systemic circulation is a prognostic indicator for many cancers (16).

Currently, the independent risk factors for bone metastatic breast cancer (BMBC) are inconsistent, and a dedicated prediction tool for BMBC is lacking. A nomogram is a reliable and convenient prognostic tool, and it is widely used in oncology prediction because of its incorporation of quantitative analysis of risk variables (17–19). However, since the prognostic nomograms of patients with MBC were created using mostly the Surveillance, Epidemiology, and End Results (SEER) database, the possibility of extending these nomograms to the Chinese population is unclear (20, 21). Chinese women have various clinicopathological and ethnic characteristics and health insurance plans. Thus, the accurate prediction of DMFS in Chinese patients with BC may improve their monitoring and treatment options. This study had two purposes: first, to identify factors associated with bone metastasis of breast cancer; and second, to establish a nomogram predicting bone metastasis of early breast cancer through clinicopathological features and hematological parameters.

## 2 Methods

### 2.1 Patient population

From 2010 to 2020, we retrospectively evaluated the relationship between hematological parameters, clinicopathological characteristics, and MBC of BC patients at the Shandong Cancer Hospital. We adopted the following inclusion criteria: (1) female patients with BC, (2) the person receiving the operation; (3) patients with available follow-up information; (4) no preoperative chemotherapy or radiotherapy (5) no blood transfusion or other anti-cancer treatment before blood examination; and (6) complete analysis of hematological parameters from admission to the day before surgery. The exclusion criteria were as follows: (1) bilateral primary BC, (2) patients with primary or occult BC (T0), (3) incomplete medical records, (4) patients with distant metastasis at the first visit, and (5) patients with autoimmune diseases or aggressive viral infections (such as HIV or hepatitis). A total of 1042 patients were included in the study, and 730 were randomly assigned to the training set and 312 to the validation set.

### 2.2 Clinical variables

The following clinical and pathological information were obtained from medical records: surgery, age at diagnosis, LVI, body mass index (BMI), pathological tumor grade, pathological (pT) stage (T1, T2, T3), pathological node (pN) stage (N0, N1, N2, N3), ER status, PR status, HER2 status, molecular subtype (Luminal A, Luminal B(HER2-), Luminal B(HER2+), HER2-enriched, TNBC), clinical treatment, timing of bone metastases, number of bone metastasis sites and follow-up. Oligometastases of breast cancer bone metastases are usually defined as the number of metastatic sites ≤3 and no distant visceral metastases. Platelet, neutrophil, monocyte, and lymphocyte counts are examples of obtained hematological parameters. The correlation of hematological indicators was described using the following terminology: NLR, MLR, PLR, SII, and lymphocyte count/monocyte count ratio (LMR). These parameters were taken from the last hematological examination before surgery. Molecular subtype was performed according to the 2021 St. Gallen Breast Cancer Guidelines. We obtained postoperative pathological specimens from the patients and performed immunohistochemical analysis. All analyses were performed in the ancillary department of our institution.

### 2.3 Statistical analysis

The ideal cut off levels for NLR, MLR, PLR, SII, and LMR were identified using ROC curve analysis. A ratio of 7:3 was used to divide all patients into training and verification sets. In univariate analysis, categorical variables were compared using the Pearson chi-square test and Fisher’s exact probability test, while quantitative data with normal or abnormal distributions were subjected to the t-test or Wilcoxon rank test. Logistic regression was used to perform a multivariate analysis, and factors with statistical significance (*P*< 0.05) were considered. The prediction model was then created using binary logistic regression and significant variables from multivariate analysis. In order to identify independent risk factors for BMBC, a nomogram of BMBC was established based on multivariate logistic regression (*P*< 0.05). ROC curves were used to assess the area under the curve (AUC) of the nomogram. An AUC value closer to 1 indicates more accurate risk prediction. Calibration curves of the prediction model were drawn to assess the compatibility between predicted BMBC probability and observation probability. BMBC probability is displayed on the y-axis of the calibration curve, while e x-axis of the calibration curve shows the BMBC probability predicted by the training and verification sets. R4.2.2 and SPSS version 26.0 (SPSS, Chicago, Illinois, USA) were used to analyze the data (R Statistical Computation Project, www.r-Project.org). Finally, a risk classification model was developed based on the overall nomogram score of each patient. To categorize the patients into low- and high-risk groups, we used the nomogram’s median risk score as the dividing line. BMBC probabilities for various risk categories were compared using the Kaplan-Meier method. *P*< 0.05 was set as the threshold for statistical significance.

## 3 Results

### 3.1 Patient characteristics

**Figure 1:**
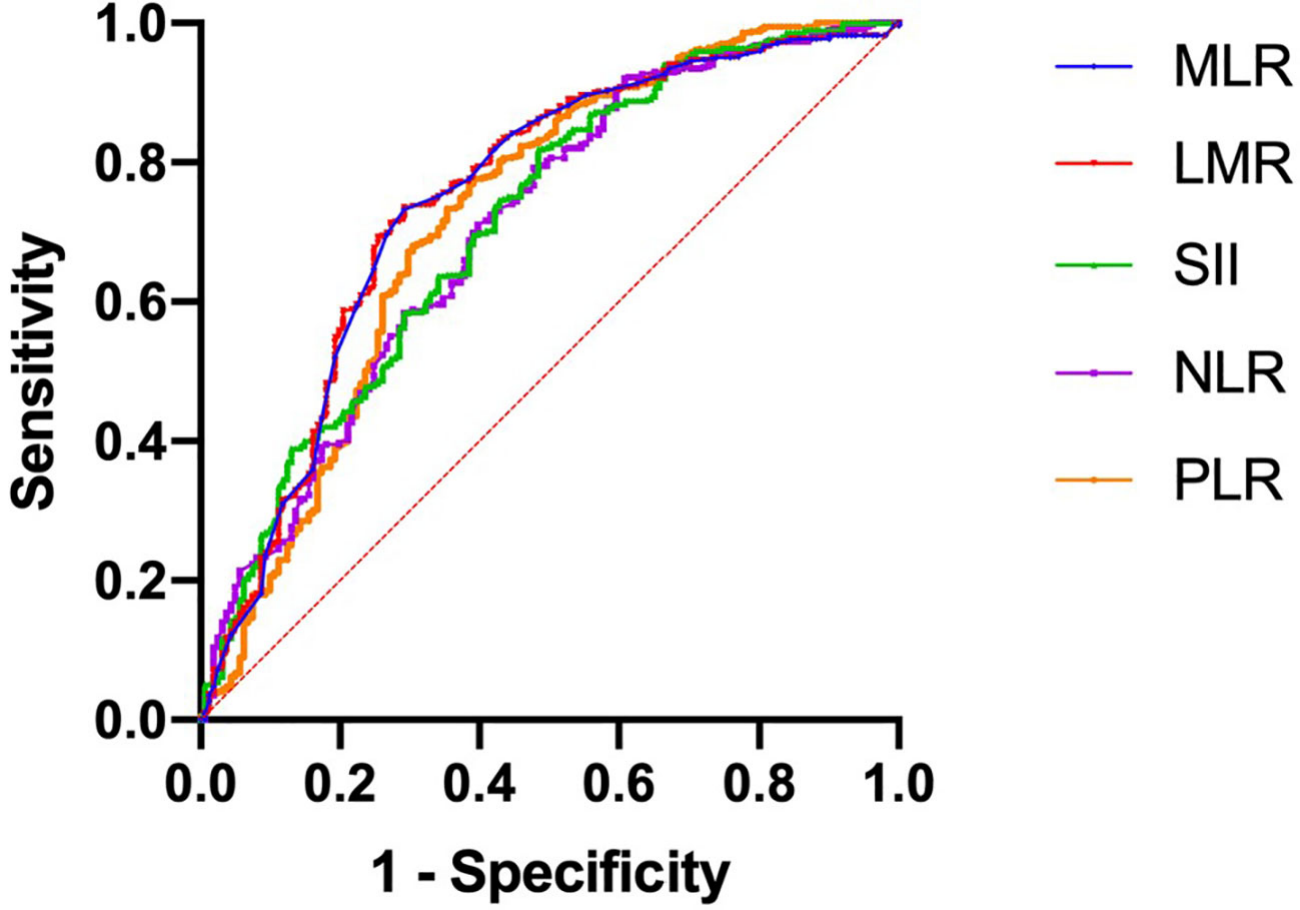
Receiver operating characteristic curves were generated to evaluate the cutoff value of the hematological parameters.

A total of 1043 women with BC between 2010 and 2020 were enrolled (training set, 730 patients; validation set, 312 patients). A median follow-up time of 73 months was observed in the training set and 71 months in the validation set. As shown in **Table 1**, the training and validation sets have the following baseline clinical characteristics. In the training set, the median age at diagnosis was 45 years (range, 18–76 years), and the median BMI was 24.02 (range 13.93 to 34.77). Overall, 668 (91.51%) patients underwent modified radical mastectomy, and 62 (8.49%) underwent breast-conserving surgery. Vascular tumor thrombus infiltration was observed in 75 (10.27%) patients. In the T stage, 461 (63.15%), 247 (33.84%), and 22 (3.01%) cases corresponded to T1, T2, and T3, respectively. In the N stage, N0, N1, N2, and N3 occurred in 449 (61.51%), 179 (24.52%), 52 (7.12%), and 50 (6.85%) cases, respectively. Regarding molecular subtype, LuminalB(HER2-)patients accounted for the highest proportion (40.82%), followed by LuminalA patients (27.67%), LuminalB (HER2+) patients (14.11%), TNBC patients (9.45%) and HER2-enriched patients (7.95%). ROC and cutoff values for MLR, LMR, SII, NLR, and PLR in patients with BC after surgery are presented in **Figure 1** and **Table 2**. The best cutoff point was used for subsequent statistical analysis.

**Table 1:** Demographic and clinicopathologic characteristics of the cohort with BMBC.

Variables	Total cohort (N=1,042)	Training cohort(N=730)		Validation cohort(N=312)		*P* Value
		Total	BCBM	Total	BCBM	
**Age**						<0.001
≤45	529(51.8%)	370	99(26.8%)	159	41(25.8%)	
>45	513(49.2%)	360	66(18.3%)	153	32(20.9%)	
**BMI**						0.108
≤24	525(50.4%)	359	65(18.1%)	166	32(19.3%)	
>24	517(49.6%)	371	100(27.0%)	146	41(28.1%)	
**Surgery**						0.754
MRM	952(91.4%)	668	150(22.5%)	284	66(23.2%)	
BCS	90(8.6%)	62	15(24.2%)	28	7(25%)	
**LVI**						<0.001
Yes	115(11.0%)	75	46(61.3%)	40	26(65.0%)	
No	927(89.0%)	655	119(18.2%)	272	47(17.3%)	
**Grade**						<0.001
I	88(8.4%)	62	7(11.3%)	26	3(11.5%)	
II	584(56.1%)	413	60(14.5%)	171	30(17.5%)	
III	370(35.5%)	255	98(38.4%)	115	40(34.8%)	
**pT stage**						<0.001
T1	645(61.9%)	461	53(11.5%)	184	21(11.4%)	
T2	361(34.6%)	247	91(36.8%)	114	40(35.1%)	
T3	36(3.5%)	22	21(95.5%)	14	12(85.7%)	
**pN stage**						<0.001
N0	652(62.6%)	449	45(10.0%)	203	20(9.9%)	
N1	244(23.4%)	179	49(27.4%)	65	21(32.3%)	
N2	72(6.9%)	52	33(63.5%)	20	14(70.0%)	
N3	74(7.1%)	50	38(76.0%)	24	18(75.0%)	
**ER**						0.668
Positive	826(79.3%)	575	132(23.0%)	251	60(23.9%)	
Negative	216(20.7%)	155	33(21.3%)	61	13(21.3%)	
**PR**						0.769
Positive	754(72.4%)	533	119(22.3%)	221	51(23.1%)	
Negative	288(27.6%)	197	46(25.7%)	91	22(24.2%)	
**Ki67**						0.002
≤20	372(35.7%)	265	43(16.2%)	107	14(13.1%)	
>20	670(64.3%)	465	122(26.2%)	205	59(28.8%)	
**Her-2**						<0.001
Positive	402(38.6%)	161	53(32.9%)	71	25(35.2%)	
Negative	640(61.4%)	569	112(19.7%)	241	48(19.9%)	
**Molecular subtype**						0.001
Luminal A	285(27.4%)	202	31(15.3%)	83	9(10.8%)	
Luminal B(HER2-)	426(40.9%)	298	67(34.0%)	128	32(25.0%)	
Luminal B(HER2+)	153(14.7%)	103	39(37.9%)	50	20(40.0%)	
HER2-enriched	79(7.6%)	58	14(24.1%)	21	5(23.8%)	
TNBC	99(9.5%)	69	14(20.3%)	30	7(23.3%)	
**Chemotherapy**						0.749
Yes	826(79.3%)	582	133(22.9%)	244	56(23.0%)	
No	216(20.7%)	148	32(21.6%)	68	17(25.0%)	
**Radiotherapy**						0.001
Yes	594(57.0%)	421	76(18.1%)	173	34(19.7%)	
No	448(43.0%)	309	89(28.8%)	139	39(28.1%)	
**Bone metastasis**						
Oligometastases	169(71.0%)	–	117(70.9%)	–	52(71.2%)	
Multifocal metastasis	69(29.0%)	–	48(28.1%)	–	21(28.8%)	

HER2, Human epidermal growth factor receptor 2; MRM, Modified radical mastectomy; BCS, Breast-Conserving Surgery.

**Table 2:** The optimal cutoff point for BMBC.

Variables	AUC	Cutoff point	*P* Value
MLR	0.7464	0.225	<0.001
LMR	0.7468	4.435	<0.001
SII	0.7113	576.26	<0.001
NLR	0.7053	2.33	<0.001
PLR	0.7218	167.07	<0.001

NLR, neutrophil count/lymphocyte count ratio; MLR, monocyte count/lymphocyte count ratio; PLR, platelet count/lymphocyte count ratio; SII, platelet count * neutrophil count/lymphocyte count ratio; PLR, lymphocyte count/monocyte count ratio; AUC, area under the curve.

### 3.2 Univariate and multivariate analysis

In univariate analysis, age at diagnosis, LVI, grade, pT stage, pN stage, molecular subtype, PLR, NLR, SII, LMR, MLR, and radiotherapy were correlated with BMBC. In multivariate logistic regression analysis, age at diagnosis, LVI, pT stage, pN stage, molecular subtype, PLR, SII and radiotherapy were found to be independent predictors. These variables were used to construct the nomogram. The results of the univariate and multivariate analysis are shown in **Table 3**.

**Table 3:** Univariate and multivariate regression analysis based on variables for BMBC.

Variables	Univariate Analysis		Multivariate Analysis	
	OR(95%CI)	*P* Value	OR(95%CI)	*P* Value
**Age**	0.957 (0.938, 0.979)	<0.001	0.945 (0.918, 0.972)	<0.001
**BMI**	1.045 (0.990, 1.102)	0.108	–	–
Surgery				
MRM	Reference		–	–
BCS	1.102(0.599, 2.026)	0.754	–	–
LVI				
No	Reference		Reference	
Yes	7.145(4.310, 11.843)	<0.001	2.711 (1.202,6.112)	0.016
Grade				
I	Reference		Reference	
II	1.335(0.581, 3.071)	0.496	0.932(0.298,2.917)	0.903
III	4.904(2.147, 11.203)	<0.001	2.687(0.842,8.570)	0.095
pT stage				
T1	Reference		Reference	
T2	4.491(3.054,6.604)	<0.001	3.172(1.855,5.422)	<0.001
T3	161.660(21.308,1226.468)	<0.001	119.651(7.086,2020.265)	0.001
pN stage				
N0	Reference		Reference	
N1	3.384(2.157, 5.309)	<0.001	2.526(1.391,4.584)	0.002
N2	15.593(8.197, 29.661)	<0.001	15.868(6.422,39.207)	<0.001
N3	28.430(13.860,58.313)	<0.001	24.066(8.921,64.920)	<0.001
Molecular subtype				
Luminal A	Reference		Reference	
Luminal B (HER2-)	1.600(1.001,2.558)	0.050	0.832(0.411,1.684)	0.608
Luminal B (HER2+)	3.361(1.935,5.839)	<0.001	2.691(1.171,6.182)	0.020
HER2-enriched	1.755(0.860,3.580)	0.122	0.618(0.218,1.754)	0.366
TNBC	1.404(0.697,2.829)	0.342	0.650(0.215,1.966)	0.445
PLR				
Negative	Reference		Reference	
Positive	5.226(3.613,7.559)	<0.001	1.986(1.007,3.917)	0.048
NLR				
Negative	Reference		Reference	
Positive	4.214(2.918,6.084)	<0.001	1.003(0.504,1.998)	0.993
SII				
Negative	Reference		Reference	
Positive	4.707(3.242,6.835)	<0.001	2.225(1.011,4.90)	0.047
LMR				
Negative	Reference		Reference	
Positive	6.123(4.195,8.937)	<0.001	4.332(0.984,19.072)	0.053
MLR				
Negative	Reference		Reference	
Positive	5.961(4.057,8.757)	<0.001	0.948(0.213,4.211)	0.944
Chemotherapy				
No	Reference		–	–
Yes	1.074(0.694,1.662)	0.749	–	–
Radiotherapy				
No	Reference		Reference	
Yes	0.545(0.384,0.773)	0.001	0.248(0.143,0.428)	<0.001

### 3.3 Nomogram development and validation

**Figure 2:**
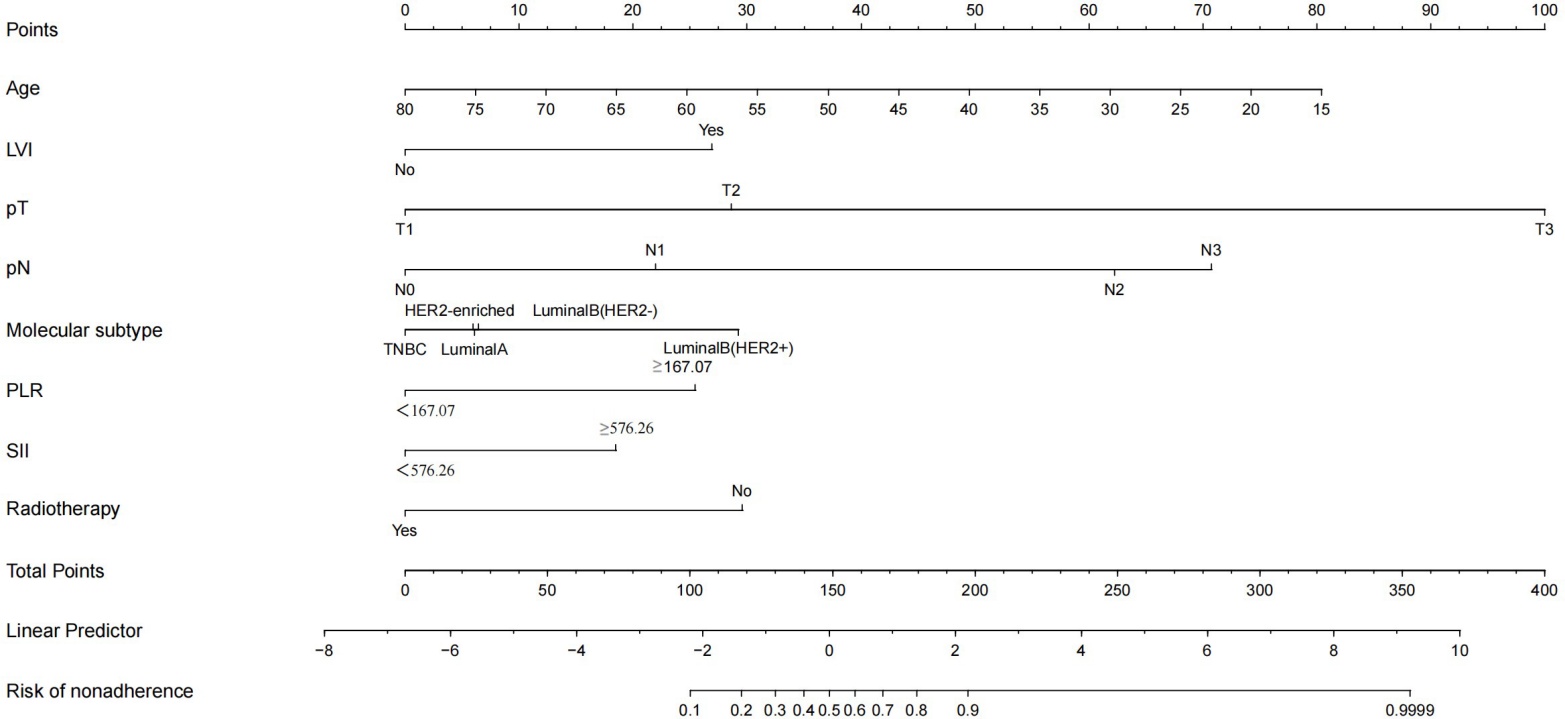
Nomogram to predict the probability of bone metastasis in a patient with non-metastatic breast cancer. Draw a line from each variable’s position to the point axis; calculate the points of different variables and add them to generate a total score, and convert them into BMBC prediction probability according to the nomogram.

**Figure 3:**
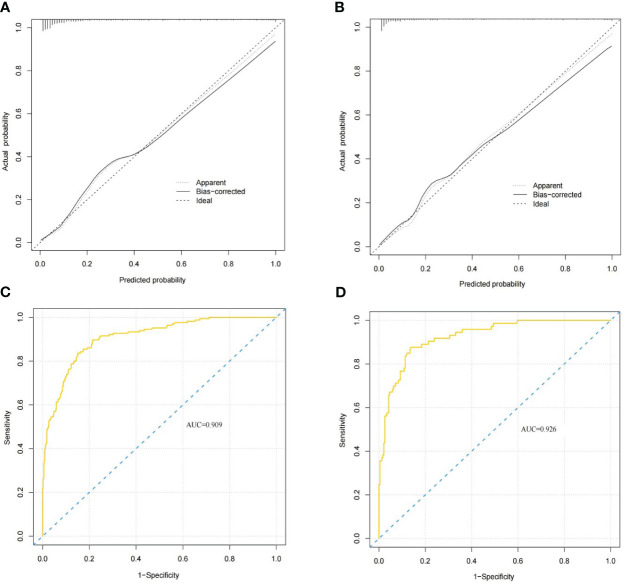
BMBC nomogram evaluation **(A–D)**. Nomogram calibration curves for the training set **(A)** and validation set **(B)**. Receiver operating characteristic curves for the training set **(C)** and validation set nomograms **(D)**. The areas under the curves for the training cohort and the validation cohort were 0.909 and 0.926, respectively.

**Figure 4:**
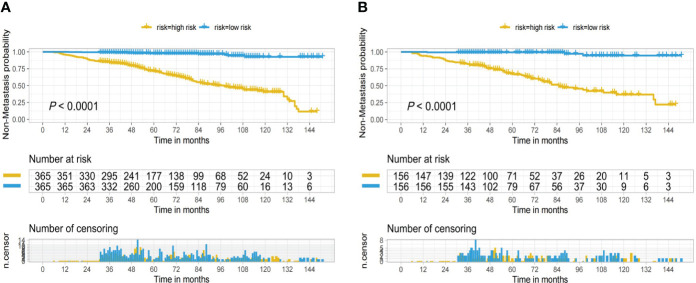
Kaplan-Meier curves for low and high risk bone metastatic cancer breast based on the nomogram of the training set **(A)** and validation set **(B)**.

**Figure 5:**
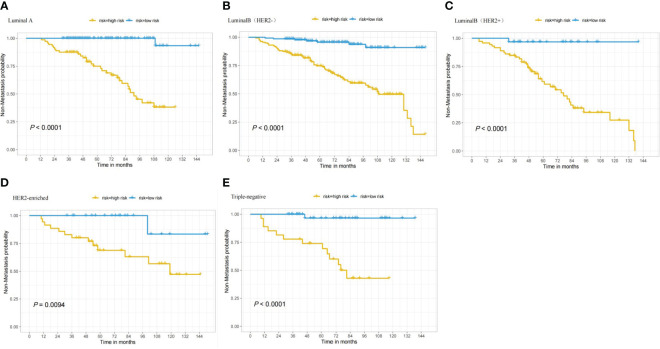
Based on patients with various nomogram subtypes, the Kaplan-Meier curve shows low and high risk BMBC. **(A)** DMFS in patients who have LuminalA tumors; **(B)** DMFS in patients who have LuminalB(HER2-) tumors; **(C)** DMFS in patients who have LuminalB(HER2+) tumors; **(D)** DMFS in patients who have HER2-enriched tumors; and **(E)** DMFS in patients who have TNBC tumors.

In the nomogram, the scores for each factor on the coordinate axis were added to obtain the total BMBC recurrence risk score (**Figure 2**). In our study, age, N stage, and T stage had the greatest impact on the prediction results, followed by radiotherapy, molecular subtype, LVI, PLR and SII. The nomogram of BMBC shows excellent prediction accuracy. We validated our model using calibration curves, and both the training set (**Figure 3A**) and the verification set (**Figure 3B**) show that our model has good calibration. Subsequently, we drew the ROC curve of the prediction probability and calculated the AUC values of the development and verification groups. The AUC values in the training (**Figure 3C**) and verification (**Figure 3D**) sets were 0.909 and 0.926, respectively. Thus, the nomogram predictions and actual BMBC are in good agreement. According to the nomogram, all patients were divided into two risk levels: a low-risk group (*≤95.9*), and a high-risk group (*>95.9*). In addition, Kaplan Meier analysis showed that risk stratification based on nomograms could accurately distinguish BMBC with different DMFSs. The low-risk patients had significantly better DMFS than the high-risk patients (**Figure 4**). Even when stratified analyzed for different subtypes of patients, the Kaplan-Meier curve showed significant differences in metastasis rates between the high-risk and low-risk groups (**Figure 5**).

## 4 Discussion

This study aimed to identify risk factors associated with BC with bone metastasis and establish a nomogram predicting bone metastasis of early BC through clinicopathological features and hematological parameters. We found that age at diagnosis, LVI, grade, pT stage, pN stage, molecular subtype, PLR, NLR, SII, LMR, MLR, and radiotherapy were correlated with BMBC. Furthermore, age, LVI, pT stage, pN stage, molecular subtype, PLR, SII and radiotherapy were found to be independent predictors of BMBC by multivariate regression analysis. Although some tools, such as the AJCC-TNM staging system, can be used to predict the prognosis of patients with BC, they ignore some important variables, such as hematological indicators (22). In addition, the AJCC-TNM staging system was developed using data obtained from Western women who differ from Chinese women in terms of their biological characteristics. Therefore, it is necessary to develop a more comprehensive model that can better predict individualized patient outcomes to improve the predictive power of BMBC.

Through multivariate analysis, we identified independent risk factors for patients with BMBC. Previous studies have also shown that age, tumor size, tumor grade, and molecular subtype may be risk factors for MBC, which is consistent with the results of our study (21, 23–25). Our study indicates that pN stage, which reflects the status of the axillary lymph nodes, is an important independent risk factor for BMBC with a significant prognostic impact. Patients with advanced pN stage may still have a risk of persistent residual cancer cells after systemic treatment, and the tumor has the characteristics of adjacent lymph node metastasis, resulting in a higher risk of recurrence and metastasis. In our study, LuminalB (HER2+) patients were more likely to develop bone metastasis. On the one hand, the incidence of bone metastases in HR+ patients was higher than in HR−patients, which is consistent with previous studies by Shi and Xiao et al. (26, 27). On the other hand, the distant metastasis-free interval was shorter in breast cancer HER2+ patients. LuminalB (HER2+)tumors have a better prognosis than other subtypes (27), and we found that patients with these subtypes were more prone to bone metastases. In addition, independent risk factors for BMBC, such as young age (23, 24), advanced T stage (24, 25), positive LVI (28), and no radiation therapy (29), were consistent with those reported for other breast cancers.

With the development of research on the tumor microenvironment, an important intermediary exists between the inflammatory response and cancer progression (30, 31). Studies have shown that massive release of inflammatory factors in the circulatory system adversely affects the prognosis of patients with BC (32). Systemic inflammatory factors increase cancer cell invasion and proliferation, thereby promoting tumor growth and progression. We further analyzed the correlations between PLR, NLR, SII, LMR, MLR, and BMBC. The results showed that the incidence of bone metastases was higher in patients with high SII and PLR than in those with low SII and PLR. The data validated the value of the SII and PLR as BMBC prognostic biomarkers, and this was consistent with the results of previous studies (33, 34). Moreover, changes in inflammatory factors can promote tumor growth and metastasis (35, 36). During tumor progression, platelets promote angiogenesis, mainly by adhering to tumor vessels and releasing granules containing platelet-derived endothelial growth factor (37). In the local tumor inflammatory environment, greater infiltration of neutrophils and platelets and less infiltration of lymphocytes can be observed. Lymphocytes contribute to the destruction of residual malignant tumor cells and associated micrometastases in host cells, and play an important role in the immune regulation of host cells (38). Studies have shown that the markers we used such as SII and PLR may be considered risk factors for BMBC. The SII takes into account the combined effects of platelet, neutrophil, and lymphocyte counts. In a study of patients with BC, Zhang et al. found that an elevated SII predicted lower survival outcomes and was correlated with clinicopathological features indicative of tumor progression (39). Another meta-analysis of the prognostic value of PLR in BC showed that PLR is an effective prognostic biomarker (33). This is consistent with our study findings.

To the best of our knowledge, this study is the first to simultaneously compare clinicopathological features and inflammatory markers of BC (PLR, NLR, SII, LMR, and MLR) and establish a BMBC nomogram that includes SII and PLR to predict the incidence of bone metastases in patients. The nomogram we developed showed a satisfactory predictive effect. Unlike the TNM staging system model, in which the C-indices range from 0.678 to 0.775 (23–25), our model achieves more accurate predictions and is economical and convenient. However, this study has some limitations. It is a single-center retrospective study; thus, there are some uncertain biases, and the study has not been validated in other centers or databases. Therefore, the influencing factors and prediction models of BMBC require further verification. Despite these limitations, we found that age at diagnosis, LVI, pT stage, pN stage, molecular subtype, PLR, SII, and radiotherapy were significantly associated with BMBC. Furthermore, young age, positive LVI, late pT stage, late pN stage, LuminalB (HER2+) subtype, high PLR, high SII, and no radiotherapy were predisposing factors for the incidence of bone metastases. These findings can be used to identify high-risk patients to improve follow-up plans by raising early suspicion of relapse, in addition to helping clinicians optimize BMBC treatment strategies and surveillance plans to provide better treatment for patients.

## Data availability statement

The raw data supporting the conclusions of this article will be made available by the authors, without undue reservation.

## Ethics statement

Written informed consent was obtained from the individual(s) for the publication of any potentially identifiable images or data included in this article.

## Author contributions

ZY designed this research. ZT and CL conducted analyses of the statistics and drafted the manuscript. XW, HS, and PZ carried out collection of data and processed the figures or tables. All of the authors reviewed the manuscript. All authors contributed to the article and approved the submitted version.
